# Growth Ring Orientation Effects in Transverse Softwood Fracture

**DOI:** 10.3390/ma14195755

**Published:** 2021-10-02

**Authors:** Parinaz Belalpour Dastjerdi, Eric N. Landis

**Affiliations:** Civil & Environmental Engineering, University of Maine, 5711 Boardman Hall, Orono, ME 04469, USA; parinaz.belalpour@maine.edu

**Keywords:** fracture energy, crack resistance, wood fracture, end grain orientation

## Abstract

In this study, the fracture mechanics of eastern spruce were characterized in relation to end-grain orientation. Compact tension-type specimens with small pre-formed cracks were prepared such that grain angle varied relative to the load axis. Specimens were loaded under crack mouth opening displacement (CMOD) control as to maintain stable crack growth. Specimen fracture was characterized using both *R*-curve and bulk fracture energy approaches. The results showed that under a RT grain orientation, as well as grain deviations up to about 40${}^{\circ }$, cracks will follow a path of least resistance in an earlywood region. As the grain angle exceeds 40${}^{\circ }$, the crack will initially move macroscopically in the direction of maximum strain energy release rate, which extends in the direction of the pre-crack, but locally meanders through earlywood and latewood regions before settling once again in an earlywood region. At 45${}^{\circ }$, however, the macroscopic crack takes a turn and follows a straight radial path. The results further show that RT fracture is macroscopically stable, while TR fracture is unstable. None of the end-grain fracture orientations showed rising *R*-curve behavior, suggesting that there is not a traditional fracture process zone in this orientation.

## 1. Introduction

Wood is a heterogeneous, anisotropic, cellular material. It has been among the most-used structural materials for several millennia due to its excellent structural properties, its abundance, and its ease of use. Our vast experience with wood as a structural material has taught us appropriate and inappropriate ways for wood to carry loads. Specifically, any significant axial or flexural loads should be carried parallel to the axis of the wood grain, while stresses perpendicular to the grain axis (particularly tension) are to be avoided. The literature on engineering properties of wood is therefore quite rich with studies focused on stress states parallel to the grain axis, with summaries of mechanical properties in general [1] and fracture properties in particular [2] found in published texts. The emergence of mass timber in the form of cross-laminated timber (CLT) over the past 20 years, however, has necessitated a closer look at strength properties in a plane perpendicular to the grain axis. In particular, for CLT cross laminations, so-called rolling shear [3,4,5] is among the dominant failure modes for certain CLT layups. Hence, the motivation for the work described in this paper is to better understand strength and fracture in the plane perpendicular to the grain axis of the wood. Specifically, we seek to characterize crack propagation of wood relative to the orientation of the growth rings. An improved understanding of this type of fracture can drive the formulation of improved models for performance prediction of mass timber structures.

**Figure 1:**
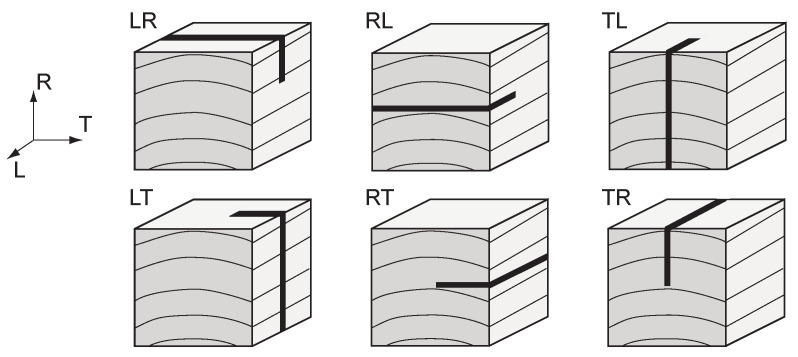
Illustrations of different possible crack orientations relative to wood grain.

Most studies in wood fracture have focused on crack propagation along the grain. In wood technology, the convention in wood is to use LRT notation for the direction relative to the growth rings (L = longitudinal, R = radial, and T = tangential direction). For defining crack propagation, a two-letter convention is used in which the first letter defines the crack plane orientation (crack normal vector), and the second letter defines the direction of propagation. The possible combinations are illustrated in Figure 1. Using this notation, we would say a majority of wood fracture research has focused on the LR and LT directions rather than TR and RT directions. The LR/LT focus has generally been justified by the afore-mentioned preference for carrying load such that the primary stress axis is oriented along the grain [2]. It should be noted that RL and TL orientations have also not had much attention from a fracture mechanics perspective, since wood loaded longitudinally tends to fracture both across the grain (RL and TL) and along the grain (LR and LT) [6,7,8]. Hence, the primary focus on LR and LT. Other such examples include Dourado et al. [9], who investigated wood behavior under mode I loading in a double cantilever beam test to determine the fracture energy in the RL system. In addition, Fonselius [10] determined the wood fracture toughness values in modes I and II. They showed that in mode I, the orientation of the specimen is important, but for mode II, the density of the wood material is the most important factor.

The importance of crack propagation increases when there are stresses perpendicular to the grain. Tukiainen and Hughes [11] investigated the fracture behavior of spruce and birch wood in compact tension in the RT direction under air-dried and green conditions. They implemented image capture and optical microscopy to demonstrate crack propagation and local deformations. Intercellular fracture appeared to be the main mechanism in both wet birch and spruce. Keunecke et al. [12] concluded that micro-cracks were likely to form in dry spruce specimens under mode I in the RT direction. They explained that this phenomenon affects the fracture toughness, but the effect was immeasurable. Stanzl et al. [13] studied wood fracture in yew and spruce wood in the TR crack propagation system. They implemented a micro-wedge splitting device for loading in the TR direction. Their study demonstrated that the higher crack propagation resistance was attributed mainly to the different cell geometries and fiber angles to the load axis of the reaction. Murata et al. [14] investigated cherry and walnut wood in TR and RT systems. They determined mode I and mode II fracture toughness through Arcan tests. Results showed that in mode II, the crack propagated in the direction normal to the shear plane, suggesting a dependence on material morphology.

Dill-Langer et al. [15] identified two different damage mechanisms of softwood loaded in tension perpendicular to the grain (both TR and RT propagation system). They reported cell wall rupture of earlywood as dominant mechanisms when the crack propagates in T direction (RT propagation system) and they observed debonding of wood fibers in the case of crack propagation in R direction (TR propagation system). Based on their results, the fiber rupture mechanism showed unstable crack, due to brittle behavior of wood in the RT system. Fruhmann et al. [16] also identified two main fracture mechanisms in the samples loaded in the TR propagation system; cell wall rupture and cell wall separation. They showed that cell wall rupture was the dominant form of damage in spruce earlywood. However, cell wall separation appeared mainly in spruce and beech latewood. They determined density is an important factor for damage mode. Conrad et al. presented three various crack paths; crack propagates by cell fracture or cell separation or it could be arrested at a vessel and split the wall of a vessel [17].

Given this previous work on cross-grain fracture, several important questions remain. First, is there any evidence of toughening mechanisms at work during RT and TR fracture, and the related question, is this crack propagation macroscopically stable? Second, is fracture and the resulting crack path affected by the transition between RT and TR crack orientation? As detailed below, we attempted to answer these questions through closed-loop controlled tensile fracture tests of specimens with different cross-grain orientation relative to the tensile stress. Fracture was characterized using an *R*-curve approach as well as a bulk fracture energy approach.

## 2. Materials and Methods

### 2.1. Wood Specimens and Preparation

The material used for manufacturing the samples was commercially available Eastern spruce (Picea rubens), species group spruce-pine-fir (SPF)s No. 2 or better). Samples were cut from the same stock of boards as Gardner et al. [18], which had an average density of 461 kg/m${}^{3}$ at a moisture content of 11.1%. The average annual ring width varied between 2 mm to 3 mm in earlywood and 0.5 mm to 2 mm in latewood.

**Figure 2:**
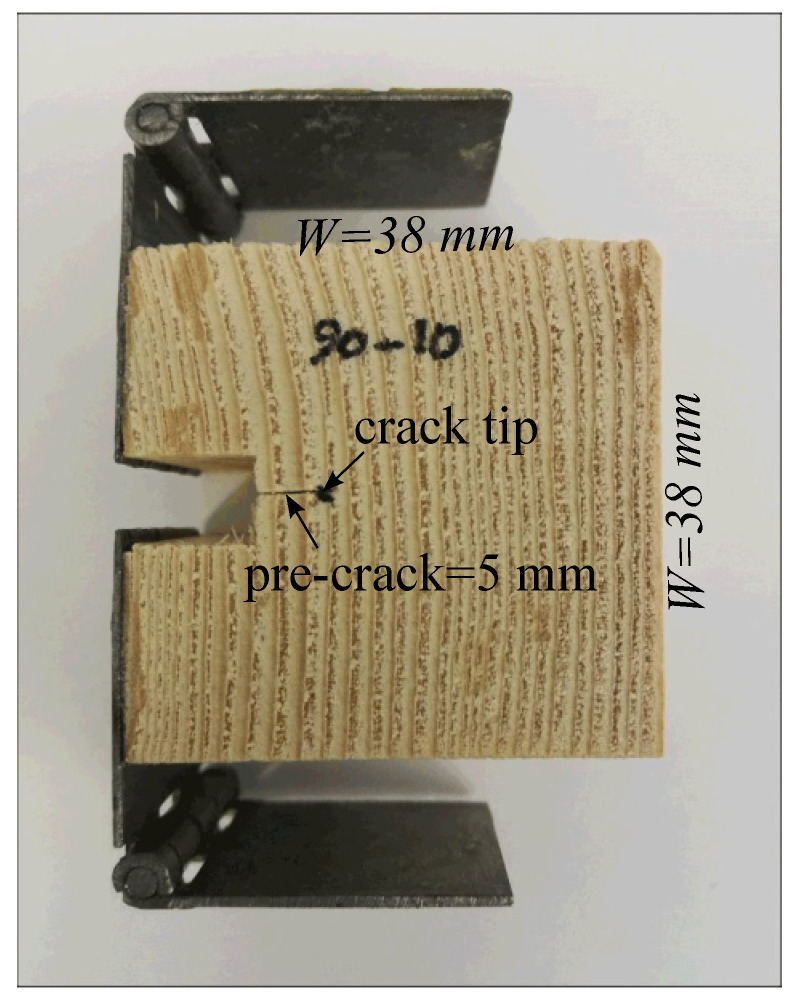
Illustration of typical compact tension specimen geometry.

**Figure 3:**
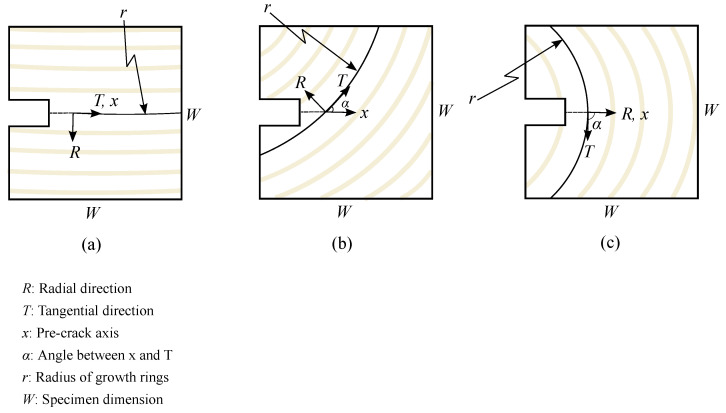
Orientation of wood end-grain, (**a**) $\alpha =0^{\circ }$ (RT), (**b**) $\alpha =45^{\circ }$ and (**c**) $\alpha =90^{\circ }$ (TR).

Specimens were cut in a compact tension geometry as illustrated in Figure 2. Specimen dimensions were 38 mm × 38 mm × 25 mm, with a 10 mm × 6 mm notch. A 5 mm-long pre-crack was cut into each specimen using a fine-edged razor so that each specimen would start with the same initial crack geometry. With respect to ring geometry, an end-grain orientation convention was adopted as illustrated in Figure 3. Here, the end-grain orientation is defined by an angle α, which is defined as the angle between the initial crack extension direction (*x*) and the tangential direction of the growth ring. Specimens were cut with α raging from 0 to 90${}^{\circ }$. In this scheme, $\alpha =0^{\circ }$ corresponds to RT, and $\alpha =90^{\circ }$ corresponds to TR crack orientations as defined in Figure 1. *r* was used to define the radius of the growth rings, which for these specimens ranged between 50 to 85 mm.

Hinged steel tabs were glued to the notched ends of the specimens to be used as grips for tension testing, as well as a clean spot onto which clip gage could be mounted for measuring crack mouth opening displacement (CMOD). An ethyl cyanoacrylate adhesive was used to bond the steel to the wood. The adhesive layer was very thin, the stiffness of the adhesive is very high, and the stresses on the bond were very low, such that any deformation of the adhesive layer would negligible in the CMOD measurement.

### 2.2. Experimental Procedure

**Figure 4:**
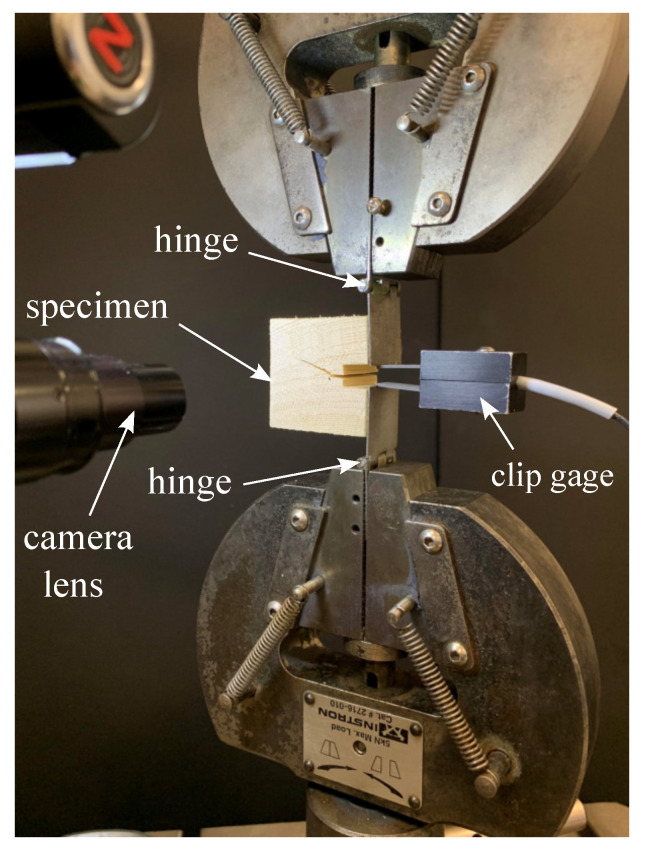
Photograph of test set-up, illustrating specimen under load.

Tension tests were conducted in a 5 kN servo-hydraulic load frame, using crack mouth opening displacement (CMOD) as the control mode. Specimens were mounted in the frame as illustrated in Figure 4. The specimens were loaded such that the CMOD increased at a rate of 0.35 mm/min. It is important to emphasize that CMOD control produces a stable crack growth condition due to a decreasing strain energy release rate function of crack length. Thus any crack instabilities measured during the test must be due to varying crack resistance rather than test procedure. Load was measured with an in-line load cell, and all load and CMOD data was recorded at 5 samples per second. Specimens were loaded until the CMOD reached 2.5 mm, or the load dropped to less than 10% of its peak.

In addition to load and CMOD data, a Nikon D750 digital camera with 6000 × 4000 pixel resolution was attached to an Infinity InfiniVar microscope lens to record images of the specimen surface, from which measurements of crack length could be made throughout the test. Images were synchronized with load frame such that the load and CMOD associated with each image could be saved. For each test, the image pixel size was determined from a calibration image, and the crack length in each subsequent image was measured using ImageJ [19,20]. In this work “crack length” is defined as the extension of the 5 mm pre-crack.

Upon test completion, specimen moisture content was measured according to ASTM D4442-16 [21]. The mean moisture content of all specimens averaged 10.3%, and did not vary by more than ±1%.

A total of 70 specimens were tested at varying end-grain angle (α). Ten specimens for each grain angle (at $0^{\circ }$(RT), $35^{\circ }$, $40^{\circ }$, $45^{\circ }$, $50^{\circ }$, $60^{\circ }$, and $90^{\circ }$(TR)) were tested. The uneven increment in grain angle was done so that we could properly capture transitions in crack growth. Complete load-CMOD data was recorded for each test, as was the associated series of digital images.

### 2.3. Fracture Analysis

#### 2.3.1. *R*-Curve

**Figure 5:**
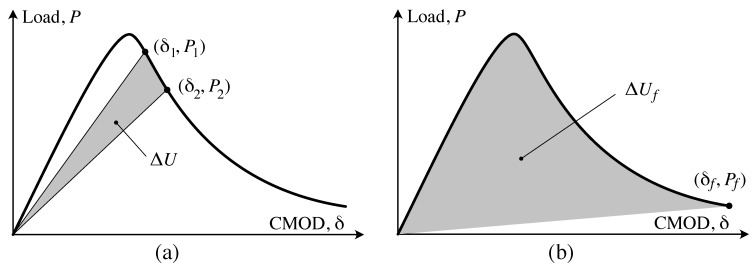
Quantities used to calculate fracture parameters. (**a**) ΔU for *R*, (**b**) $\Delta U_{f}$ for $G_{f}$.

As the goal of this work was to characterize fracture properties of wood across the grain, energy-based fracture parameters were determined from the load-CMOD data and the digital image data. A crack resistance, or *R*-curve approach was used so that any toughening mechanisms might be revealed. *R* can simply be defined as the energy required to grow a crack. This can be defined as:

(1) $$R=\frac{\Delta U}{\Delta A}$$

where ΔU is the energy dissipated by crack growth, while ΔA is the corresponding change in crack area. ΔU can be determined directly from the load-CMOD curve. This is possible because crack growth will cause an increase in specimen compliance so that the unloading path is different from the loading path. This is illustrated in Figure 5a. Given that a crack has grown between two load-CMOD points, ($\delta_{1},P_{1}$) and ($\delta_{2},P_{2}$), the energy dissipated during that crack growth can be calculated as:

(2) $$\Delta U=\left[\int_{0}^{\delta_{2}}P\left(\delta \right)d\delta -\frac{1}{2}P_{2}\delta_{2}\right]-\left[\int_{0}^{\delta_{1}}P\left(\delta \right)d\delta -\frac{1}{2}P_{1}\delta_{1}\right]$$

Note that while a simpler formula for ΔU would be possible from the illustration shown in Figure 5. However, for real data, the post-peak response was not always monotonically decreasing. Hence, there is the need for the integral form.

Regarding the crack area, ΔA, in Equation (1), in this work we could only measure the crack length on one surface of the specimen. As a result, growth in crack area was estimated using the measured crack length, multiplied by the specimen width. That is:

(3) ΔA=bΔa

where Δa is the incremental crack length change, and *b* is the specimen thickness. Combining Equations (1)–(3) yields:

(4) $$R=\frac{\Delta U}{2b\Delta a}$$

It is important to note here that the analysis presented here assumes that there is no inelastic deformation during tension testing, and as such the unloading plot will always return to the origin, as is shown in Figure 5. In the tests described here, there was no unloading until the limiting load or CMOD were reached. While it is likely that if we unloaded a specimen before those limits were reached, the plot would not return to the origin. That said, this is not necessarily an indication of plastic deformation, as localized fracture can cause changes to the cellular structure that would block elastic recovery. Indeed, mechanisms such as bridging, microcracking, or release of internal strains could all contribute to preventing complete elastic recovery.

For complete *R*-curve analysis, *R* can be calculated for each increment of crack growth and plotted as a function of crack length, *a*.

#### 2.3.2. Fracture Energy, $G_{f}$

While *R*-curve analysis is useful for evaluating incremental crack growth and presence of toughening mechanisms, it is also useful to determine energy dissipated by fracture over a longer length scale. The analysis is nearly identical to *R*-curve analysis except that rather than looking at incremental crack growth, we consider energy dissipated by fracture during the entire test. This is illustrated in Figure 5b. Here, the entire area under the curve, less the elastic recovery, is energy dissipated during the test. Given that during the test, the crack grows a length $a_{f}$, then the total fracture energy, $G_{f}$ is defined as:

(5) $$G_{f}=\frac{U_{f}}{ba_{f}}$$

**Figure 6:**
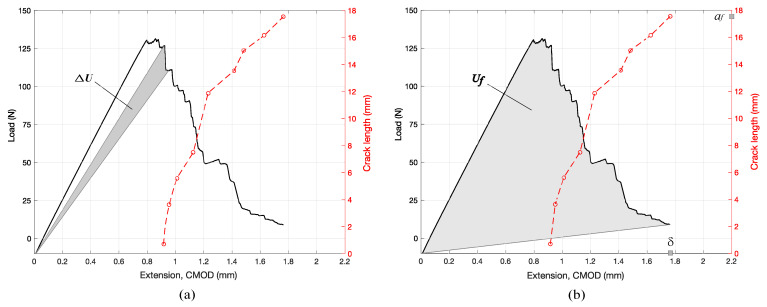
Calculation of ΔU (**a**), and $U_{f}$ (**b**).

Given $U_{f}$ as illustrated in Figure 5b, then we can write $G_{f}$ as:

(6) $$G_{f}=\frac{1}{ba_{f}}\left[\int_{0}^{\delta_{f}}P\;d\delta -\frac{1}{2}P_{f}\delta_{f}\right]$$

where $P_{f}$ and $\delta_{f}$ are the load and CMOD, respectively, at the end of the test. Figure 6 illustrates calculation *R* and $G_{f}$ for actual load-CMOD and crack length data from a RT-oriented specimen.

We should note that *R* and $G_{f}$ are related by the following:

(7) $$G_{f}=\frac{1}{a_{f}-a_{0}}\int_{a_{0}}^{a_{f}}R\left(a\right)\;da$$

where $a_{0}$ and $a_{f}$ are initial and final crack lengths, respectively. $G_{f}$ in this context may be thought of as the average value of *R* over the entire length of the crack.

## 3. Results

The primary results of interest here are the load-deformation (CMOD) responses of the different specimens, and the corresponding crack morphology. In the discussion section below, we attempt to relate the two.

**Figure 7:**
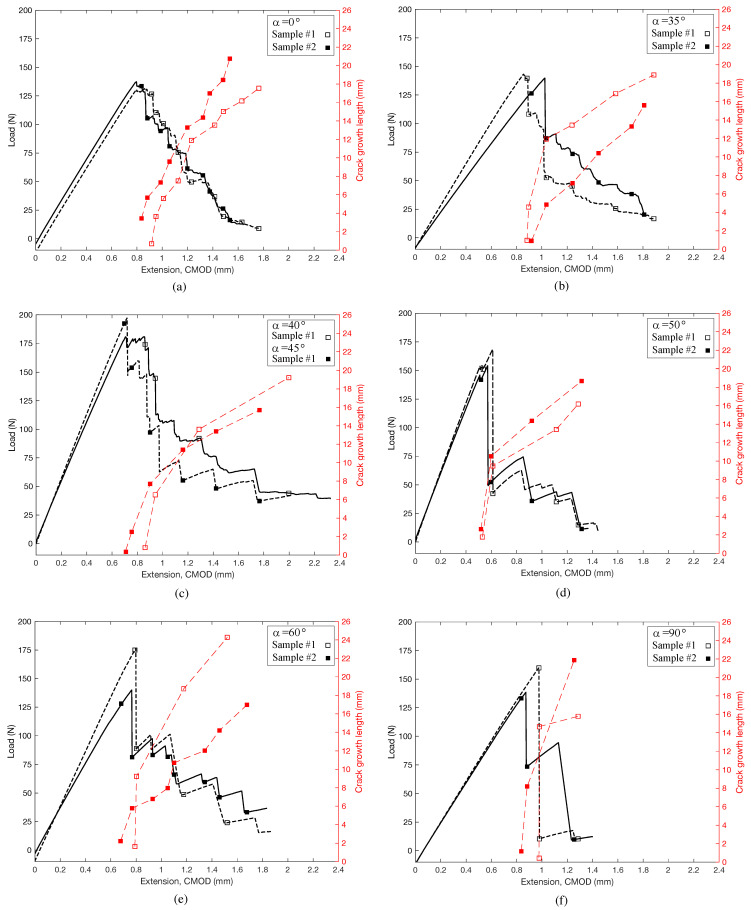
Load vs. CMOD for a range of grain angles. (**a**) $\alpha =0^{\circ }$, (**b**) $\alpha =35^{\circ }$, (**c**) $\alpha =40^{\circ }$ and $\alpha =45^{\circ }$, (**d**) $\alpha =50^{\circ }$, (**e**) $\alpha =60^{\circ }$, and (**f**) $\alpha =90^{\circ }$.

First, we consider the different load-deformation responses, as are illustrated in Figure 7 for progressively higher grain angle, α, along with corresponding crack lengths. The figure shows that all examples demonstrate a nearly perfect linear load-deformation response right up to peak load. After the peak load is reached, there are subtle but important differences in the post-peak regime. For $\alpha =0^{\circ }$ (RT orientation), the post peak exhibits a relatively constant descent, suggesting a fairly stable crack growth. This is further validated by the relatively constant rate of crack growth as a function of CMOD. Similar post-peak behavior is seen as α increases to 35${}^{\circ }$ (Figure 7b). A transition occurs between $\alpha =40^{\circ }$ and $\alpha =45^{\circ }$ (Figure 7c). For $\alpha =40^{\circ }$, we observe a slight “yield plateau” followed by several steep drops. These are then followed by a more gradual descent. The rapid drops are indicative of unstable crack growth. Note that the plot of crack length also jumps rapidly in the initial stages, but then slows with continued increase in CMOD. Recall that for CMOD control of the loading, crack growth should be stable as long as crack resistance is reasonably constant. Hence, we can conclude that any time we observe a rapid drop in load, we have an instability caused by a decrease in crack resistance. Such a decrease means that as the crack grows, less energy is required to propagate the crack further, so it propagates without any additional increase in deformation. At $\alpha =45^{\circ }$ (Figure 7c), we see a clear transition. The post-peak behavior is marked by a distinct “saw tooth” pattern in which the load drops in very discrete intervals of instability. This behavior continues for increasing α, and is most clearly pronounced for $\alpha =90^{\circ }$ (TR orientation), shown in Figure 7f. Here, the load drop is in two discrete intervals, suggesting crack growth is highly unstable, and thus the bonding mechanism perhaps has higher variability, or there are additional microstructural features, such as rays, to facilitate crack growth in the radial direction.

**Figure 8:**
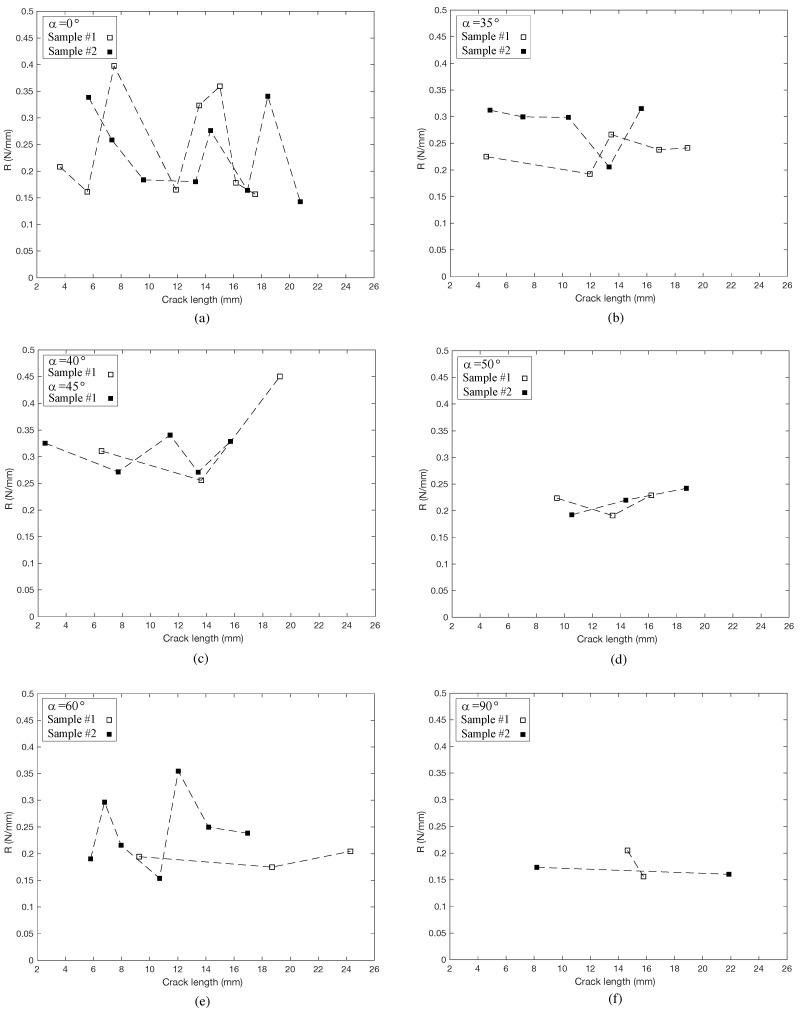
*R*-curves calculated for a range of grain angles. (**a**) $\alpha =0^{\circ }$, (**b**) $\alpha =35^{\circ }$, (**c**) $\alpha =40^{\circ }$ and $\alpha =45^{\circ }$, (**d**) $\alpha =50^{\circ }$, (**e**) $\alpha =60^{\circ }$, and (**f**) $\alpha =90^{\circ }$.

The *R*-curves calculated for these cases shed only modest light crack growth and the different load-deformation behavior. Figure 8 shows sample *R*-curves calculated using Equation (4). The curves illustrate several issues of interest. First, all specimens have a significant scatter. *R* both increases and decreases as the cracks grow, suggesting the resistance to crack growth varies considerably within a single specimen. Second, none of the *R*-curves show a statistically significant increase or decrease with crack growth. This suggests that in RT and TR fracture, as well as orientations in between, there are no significant toughening mechanisms.

**Figure 9:**
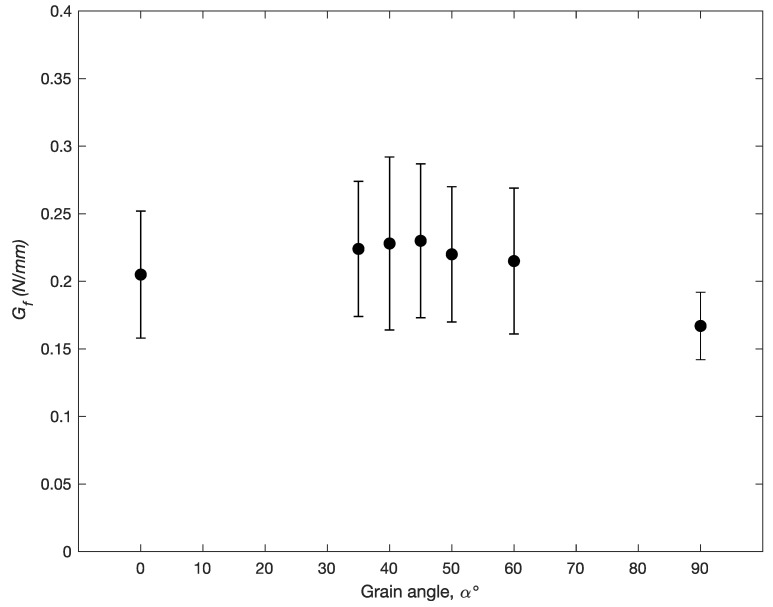
Bulk fracture energy, $G_{f}$, measured at different end-grain orientations.

The fracture energy, $G_{f}$, calculated by Equation (6) was calculated at $\alpha =0^{\circ }$, $35^{\circ }$, $40^{\circ }$, $45^{\circ }$, $50^{\circ }$, $60^{\circ }$, and $90^{\circ }$, and is shown in Figure 9. While *R* is a better measure of local fracture toughness, $G_{f}$ gives a better picture of fracture toughness over the macrocopic length of the crack. Here we see modest difference in the energy required to propagate a macrocrack among the different orientations, with $\alpha =90^{\circ }$ (TR) showing slightly lower overall toughness. There is considerable scatter among the different specimens tested within each orientation group, with COVs over 20%, which is not surprising given the *R*-curves.

**Figure 10:**
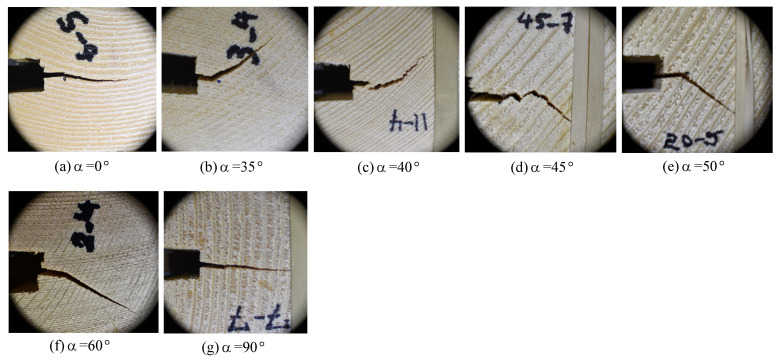
Crack growth path at different angles.

Images of crack growth for different grain orientations are shown in Figure 10. Here we see that at low α angles ($0–35^{\circ }$), the crack propagates within the earlywood band closest to the initial crack tip, and it stays in that band throughout its propagation path. The crack direction is 100% tangential. However, between 40${}^{\circ }$, and 45${}^{\circ }$, a transition takes place. At 40${}^{\circ }$ (Figure 10c), the crack still propagates in a largely tangential direction, but it jumps across several earlywood/latewood boundaries in the process. At 45${}^{\circ }$ (Figure 10d), we see a shift. After several earlywood/latewood crossings that macroscopically progress along the axis of the initial crack, the crack path turns and directly follows a radial path. By 50${}^{\circ }$, the crack path is entirely in the radial direction. This behavior continues through $\alpha =90^{\circ }$, the TR orientation.

This transition is important to note because the direction of the crack is always a trade-off between maximizing strain energy release rate (which in this configuration is in the direction of the initial crack, *x* in Figure 3), and taking the path of least resistance. Our observed transition from a tangential direction to a radial direction suggests two different paths of least resistance: one that follows a tangential path, and one that follows a radial path.

## 4. Discussion

The results presented here illustrate some of the complicated and competing mechanisms affecting softwood fracture in the transverse plane. First is the differences in fracture toughness between RT and TR crack orientations. Others ([15,16]) have examined the microstructural morphology of wood fracture and shown that tangential fracture (RT) tends to be dominated by tearing in the cell wall, while radial fracture (TR) tends to be dominated by a peeling or separation of cell walls. Both the *R*-curve analysis and the $G_{f}$ analysis show that RT has slightly higher toughness. Conrad et al. [17] suggest that lignin has a lower fracture toughness than cellulose due to a “lower molecular weight and greater polydispersity”. Given that cell separation is governed primarily by lignin fracture, our results support this. Additionally, Bodner et al. [22,23] showed that rays play an important role in wood fracture characteristics. Specifically, they showed that rays can act as stoppers for cracks propagating in the longitudinal direction (RL or TL). We suggest here that for cracks propagating in the radial direction, rays actually facilitate crack propagation by providing a path of low resistance along the ray. Such a mechanism would further support both the lower TR toughness and the added instabilities.

What our results also show is that RT and TR directions represent minima with respect to cross-grain fracture toughness. Both the *R*-curve analysis and the $G_{f}$ analysis (Figure 8 and Figure 9, respectively) show a slightly higher toughness for intermediate orientations. While these differences are not significant, with the exception of TR, we can be confident of this assertion based on the consistent shifts in crack paths with respect to grain orientation.

It should be noted that while most specimens exhibited a linear load-CMOD response up to peak load, occasionally some pre-peak cracking occurred. This manifested itself in a slight non-linearity in the load-CMOD curve prior to peak load. Such behavior is typically attributed to microcracking or other damage that is not critical with respect to macroscopic crack growth. Indeed, Stanzl-Tschett et al. [13] noted pre-peak cell wall rupture in earlywood regions followed by cell wall pealing in the post-peak for TR fracture. For the tests conducted here, the effect on *R*-curve measurements is a slightly higher initial crack resistance that falls slightly after initial macrocrack growth.

### 4.1. Crack Paths

The transition between RT and TR fracture provides an interesting case study on the trade-off between maximizing strain energy release rate and following path of least resistance. A basic tenet of fracture mechanics is that cracks grow when the strain energy release rate, *G* is equal to or greater than the crack resistance, *R*. For a compact tension specimen such as the geometry tested here, the fracture path that would release strain energy the fastest (i.e., maximum *G*) would extend in the direction of the initial crack, perpendicular to the axis of load. Indeed, the crack does this in both the RT and TR cases. As has previously been noted, however, there was a shift in the crack path as the orientation of the end-grain varied. At low end-grain α angles (closer to RT), the path of least resistance is the earlywood region. Ashby et al. [8] noted the lower density of the earlywood leads to a lower toughness. Hence, while *G* is greater for a straight crack growth, *R* is as well. But since *R* is lower for an angled crack, that becomes the direction of propagation. The crack stays in the earlywood, propagating in a tangential direction.

A transition occurs when the end-grain reaches a critical angle. As seen in Figure 10c, the crack continues to follow the earlywood, but it jumps across several latewood regions before settling into a fixed earlywood layer. Clearly the higher *G* is still “pushing” the crack to go straight. And as such, the crack has found spots where it can cut across the latewood in a way that there is a slight bias towards a straight crack, but ultimately it finds a low *R* earlywood region to follow along an angled path.

When the end-grain angle α reaches 45${}^{\circ }$, (Figure 10d), we see an interesting transition. Again, the higher *G* is pushing the crack to progress in a macroscopically straight path, but the crack does this by moving locally along earlywood, then cutting across latewood in a zig-zag manner. The crack finds local planes of weakness in alternating directions, but macroscopically, the crack extends horizontally for a short distance, maximizing *G*. But eventually the crack finds a path of least resistance (minimum *R*) in the radial direction across earlywood/latewood boundaries, rather than a tangential direction confined to earlywood. Indeed, for any end-grain angle higher than 45${}^{\circ }$, the crack propagates only in a radial direction.

This behavior implies several aspects of cross-grain fracture. First is that it confirms a lower toughness in the radial direction than the tangential direction. Since at 45${}^{\circ }$*G* is the same in either direction, *R* must be lower in the radial direction. Second, and perhaps more important, is that it confirms a higher toughness, or crack resistance, in the intermediate orientations between radial and tangential. We observe cracks growing macroscopically in the radial and tangential directions, but not in between. Thus even though the *R*-curve and $G_{f}$ calculations showed only small differences in the different directions, they are significant enough to make the crack growth directions predictable.

We should note that there is clearly a local mixed-mode component at the crack tip for any of the fracture examples shown here other than the RT and TR orientations. Thus the strain energy release rate, *G*, is made up of mode I (opening) and mode II (in-plane shear) conditions. Mode II fracture research is relatively scarce in the cross-grain (RT and TR) orientations. Murata et al. [14] found it “difficult to determine” mode II fracture toughness, as the crack rarely followed a path appropriate for a mode II condition. For this work, we simply treat the tests as macroscopically mode I, recognizing a potential contribution from a mode II resistance.

### 4.2. Crack Stability

As previously noted, a feature of the CMOD-controlled fracture tests conducted here is that they are intrinsically stable. That is, since strain energy release rate monotonically decreases as the crack grows [24], any observed instabilities in load-deformation response and the corresponding crack growth are due to local variations in *R*. This intrinsic stability can be contrasted with a single-edge-notched beam, for example [25], which requires a minimum crack length for assured stability. A quick review of the different load-deformation plots for the different end-grain orientations (Figure 6) reveal distinct behaviors with respect to crack stability. RT shows macroscopically stable crack growth, while TR shows distinct unstable crack growth. This transition occurs as the crack path shifts from macroscopically tangential to radial. As noted above, the crack growth in the tangential direction is marked by cell wall tearing. The macroscopic stability suggests that even though the RT *R*-curve showed considerable variability, the incremental instabilities in crack growth were small and that cell wall rupture properties in the earlywood were more consistent. Although it is also reasonable to suggest that each cell cavity (lumen) has the potential to act as a crack stopper, providing a microscopic level toughening mechanism. In the radial direction, which is characterized by cell separation, the a variability in fracture toughness means that anytime a crack has to overcome a localized region of high resistance, there is sufficient residual strain energy to propagate the crack through the localized region of low resistance. Furthermore, if cell separation is thought of as similar to cleavage fracture in metals, then there is no microscopic toughening mechanism to arrest the crack. Finally, if rays are present in the TR fracture plane, they can actually facilitate crack growth by producing an additional path of least resistance.

### 4.3. Toughening Mechanisms

The results further show that there is very little in the way of macroscopic toughening mechanisms at work for cracking in the transverse plane. A toughening mechanism would manifest itself through a rising *R*-curve. That is, as the crack grows, toughening mechanisms are mobilized such that continued crack growth requires incrementally higher energy. The *R*-curves calculated in this work, while showing great variability, had little in the way of trends over the length of crack growth. This is counter to what is observed in the RL or TL directions in which the crack propagates in a direction parallel to the grain. Vasic et al. [26] and Smith and Vasic [27] quantified crack bridging processes in spruce that increase the toughness as the crack progresses. In clear spruce, there seem to be no such morphological features that might produce micromechanical mechanisms for increasing toughness and indeed features such as rays may actually facilitate crack propagation. Although we have noted that at a microscopic level we have crack-stopping mechanisms during fracture in the tangential plane, at a macroscopic level, these do not lead to a so-called fracture process zone, which has the effect of increasing the energy required for additional crack growth.

## 5. Conclusions

The work described here gives us a generalized picture for cross-grain tensile fracture as a function of grain orientation. For the RT orientation and subsequent small α angles, the trade-off between maximum strain energy release rate *G*, and minimum crack resistance *R*, favor a crack propagation path that stays within an earlywood region even though an angled crack path does not maximize *G*. As the grain angle approaches 45${}^{\circ }$, the difference between *G* in the straight direction and *G* in the angled direction is high enough that the crack trends towards an extension of the original crack before it turns to a tangential direction for angles less than 45${}^{\circ }$, and a radial direction above 45${}^{\circ }$. The differences in fracture toughness between the tangential direction, which follows a path of cell wall tearing in the earlywood region, and the radial direction, which follows a path of cell wall separation, were small. However, *R*-curve analysis and gross fracture energy ($G_{f}$) analysis both showed lower toughness for cracks propagating in the radial direction, and this conclusion could also be used to explain the changes in crack propagation path with end-grain angle. Significant scatter was observed in both *R*-curve and $G_{f}$ analysis. The lack of a clear rising *R*-curve suggests little to no macroscopic toughening mechanisms. RT crack propagation was observed to be macroscopically stable, whereas TR crack propagation was unstable. While both modes show variable crack resistance, as shown by scatter in the *R*-curves, the stability of RT fracture was attributed to the cell tearing mechanism that requires the crack to propagate across the cell cavity. In such a case the cavity can potentially act as a microscopic crack stopper.

The implications of the work are that through a better understanding of cross-grain fracture mechanics, we are in a better position to model and predict performance of structural components that require wood to carry a load in that plane, such as cross-plys in CLT systems. Indeed, such an understanding could also lead to a more rational basis for cross-ply layups.

## Data Availability

The data presented in this study are available on request from the corresponding author.
